# Clinical decision system for renal cell carcinoma integrating interpretable machine learning algorithms

**DOI:** 10.3389/fsurg.2025.1588208

**Published:** 2025-11-27

**Authors:** Tianhong Zhang, Tian Tian, Yifan Zhang, Qiliang Teng, Yujie Zhang, Jun Pang, Ya Wang, Chao Yang

**Affiliations:** 1 Department of Oncology, Xi Chang People’s Hospital, Xi Chang, China; 2 Department of Radiotherapy, XuZhou Central Hospital, Xuzhou, China; 3 Department of Urology, Kidney and Urology Center, Pelvic Floor Disorders Center, The Seventh Affiliated Hospital, Sun Yat-sen University, Shenzhen, China; 4 School of Medicine, Shenzhen Campus of Sun Yat-sen University, Sun Yat-sen University, Shenzhen, China; 5 Department of Medical Administration, Xi Chang People’s Hospital, Xi Chang, China; 6 Department of Emergency, The Affiliated Hospital, Southwest Medical University, Luzhou, China

**Keywords:** kidney cancer, distal metastasis, nomogram, machine learning, predictive model

## Abstract

**Background:**

Kidney cancer is a highly heterogeneous oncologic disease with historically poor prognosis. Precise assessment of the risk of distal metastasis can facilitate risk stratification and improve prognosis for kidney cancer patients.

**Methods:**

Data from the Surveillance, Epidemiology, and End Results (SEER) database, we identified 40,527 kidney cancer patients diagnosed between 2010 and 2017 were obtained. LASSO, univariate and multivariate logistic regression analyses were employed to screen independent risk factors for distal metastasis. Six machine learning (ML) algorithms including logistic regression (LR), Naïve Bayes Classifier (NBC), Decision Tree (DT), Random Forest (RF), Gradient boosting machine (GBM) and Extreme gradient boosting (XGB), were further applied to build the predictive models. After testing with ten-fold cross-validation and receiver operating characteristic (ROC) analysis, the model with the highest area under curve (AUC) was selected as the best performing model to establish the risk predictive nomogram and web calculator.

**Results:**

In distal metastasis risk prediction, the XGB model had the best performance in both training (AUC = 0.91) and testing (AUC = 0.851) datasets among the six ML algorithms. Variables including marital status, sequence number, primary site, grade, pathological type, T-stage, N-stage, the calculated risk of XGB, surgical and radiation treatment were incorporated to establish a nomogram to predict the 1-, 3-, and 5-years survival probability. The calibration plots, decision curve analysis (DCA), ROC curves and Kaplan–Meier (KM) curves all verified the predictive utility of the nomogram.

**Conclusions:**

We established a favorable prediction for the occurrence of distal metastasis with the ML model. The nomogram based on XGB algorithm can contribute to identify high-risk patients and provide optimal clinical strategies.

## 1 Introduction

Kidney cancer is among the 10 most malignancy in USA with estimated 76,080 new cases and 13,780 deaths in 2021 (1, 2). It is a highly heterogeneous oncologic disease originating from the urinary system with historically poor prognosis (3). The 5-year survival rate was about 74% for all patients with kidney cancer, 53% for locoregional disease and 8% for metastatic disease (4, 5). Among all patients, nearly 91% cases were diagnosed at an age of 45 or older, making kidney cancer a disease of the middle- and old-aged (4). Renal-cell carcinoma (RCC) occurs in 90% kidney cancer and has diverse molecular and histologic subtypes (5).

Though more low-stage and indolent tumors were identified with the improvement of early-detection techniques, there are still one third of kidney cancer patients present with metastasis (4, 6). And nearly 25% localized RCC present with relapses in distal sites after treating with nephrectomy (7–9). The common sites of RCC metastasis are the lungs (45%), bones and brain (10). Due to the immunogenicity of metastatic RCC (mRCC), immune checkpoint inhibitors (ICIs), such as nivolumab plus ipilimumab and nivolumab monotherapy, were validated to improve the prognosis of mRCC (11, 12). Systemic therapies targeting angiogenesis and modulating immunity, such as sunitinib, bevacizumab and axitinib, have also been optimized (13–18). Nevertheless, mRCC still has a limited median survival of approximately 12 months (19). Thus, novel targets or predictive tools to predict distal metastasis and identify high-risk mRCCs are urgently required.

In the 1950s, artificial intelligence (AI) became a branch of computer science dedicated to developing algorithms to enable machines to perform complex tasks that would normally require human intelligence to accomplish. Machine learning (ML) is the main area of AI research, The integration of artificial intelligence in the medical field is developing rapidly, and there have been breakthroughs especially in the diagnosis, treatment and efficacy assessment of medicine (20).

Nomogram is a simple and practical tool widely applied in prognosis prediction. A few literatures have established nomograms to instruct clinical treatment and predict prognosis targeting metastasis from kidney cancer (21–23). While our study employed six ML algorithms including logistic regression (LR), Naïve Bayes Classifier (NBC), Decision Tree (DT), Random Forest (RF), Gradient boosting machine (GBM) and Extreme gradient boosting (XGB) to analyze the kidney cancer data from the SEER database, aimed to obtain the best ML algorithm and construct an insightful risk prediction nomogram for distal metastasis.

## 2 Methods

### 2.1 Source of data

In our study, data were extracted from the SEER database. The inclusion criteria were adopted as follows: (1) with primary kidney cancer; (2) diagnosed based on positive histology from 2010 to 2017, and the included histological subtypes including RCC, transitional cell carcinoma, clear cell adenocarcinoma, and other kidney cancer; (3) with complete survival and follow-up data until 2017; (4) age ≥18 years. Exclusion criteria were practiced as follows: (1) multiple primary malignant tumors; (2) unknown tumor characteristics and demographic information; (3) diagnosed via a death certificate; (4) with unknown distal metastasis and survival status; (5) died of causes other than kidney cancer. This research was conducted after obtaining informed consent from all patients and was approved by the ethics committee.

### 2.2 Data collection and follow-up

The included demographic features include marital status, age at diagnosis, race ethnicity and sex. We also extracted the following clinicopathological characteristics: tumor size, sequence number, primary site, grade, laterality, pathological type, TNM-staging, surgical approaches, the status of radiotherapy, chemotherapy and systemic therapy. Based on AJCC staging system, histological grades were divided into grade 1–4, corresponding to well-differentiated, moderately differentiated, poorly differentiated and undifferentiated in turn. CT examination, radionuclide bone scan and PET-CT are recommended to identify and evaluate the suspected metastatic lesions. While pathological biopsy is the gold criterion of diagnosis for the metastatic sites. The presence of distal metastasis was defined as the primary endpoint event, while survival time was the sub-endpoint event. All enrolled patients were followed up through outpatient review or telephone calls.

### 2.3 Statistical analysis

Qualitative data including demographics and clinicopathological characteristics were compared via Pearson Chi-square test. T-test were utilized to compare quantitative data on normal distribution, while Wilcoxon rank test for abnormal distribution. Six different machine learning algorithms were utilized to analyze our data: LR, NBC, DT, RF, GBM and XGB. The model having the highest AUC was regarded as the best performing model. All analyses were conducted utilizing R version 4.3.1 and SPSS version 25.0. *P* < 0.05 indicated statistical significance in all analyses.

## 3 Results

### 3.1 Baseline characteristics

**Figure 1:**
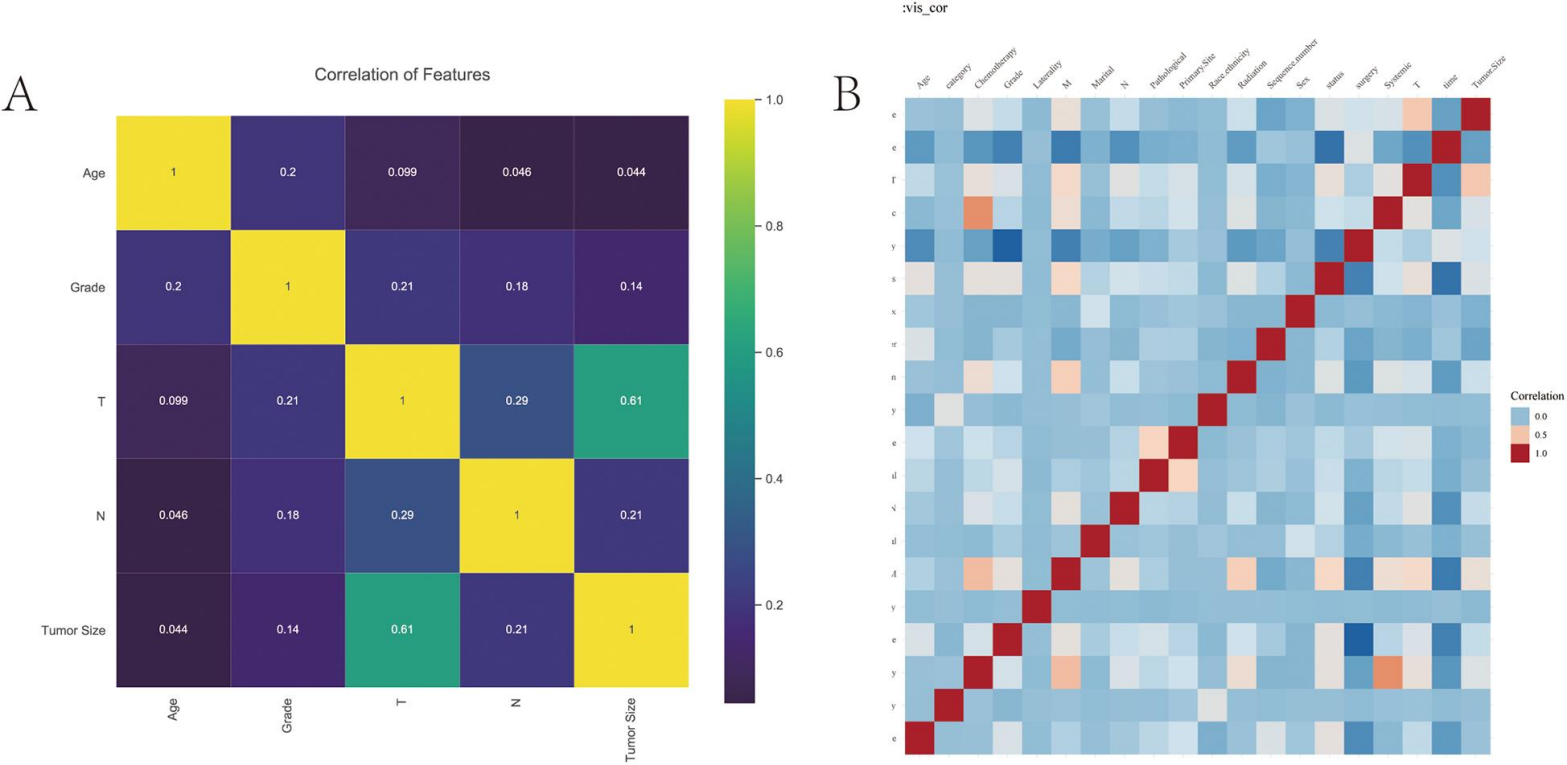
The correlation analysis of features. **(A)** The correlation of variables. Yellow indicates positive correlation and purple indicates negative correlation. **(B)** Correlation heat map.

A total of 40,527 patients from SEER database diagnosed between 2010 and 2017 were enrolled in this study. Among these patients, there were 38,525 kidney cancer patients and 2002 renal pelvis cancer patients at initial diagnosis. The detailed clinicopathological features of the whole cohort were presented in Table 1. The training group included 40,527 patients and validation group included 801 patients. And the correlation analysis of these features was displayed in Figures 1A,B.

**Table 1:** Baseline patient data for training and validation groups.

Variable	Level	Training group (*N* = 40,527)	Validation group (*N* = 801)	*P*
Marital (%)	Married	25,058 (61.83)	509 (63.55)	0.3406
	Unmarried	15,469 (38.17)	292 (36.45)	
Age (median [IQR])	NA	64.000 [55.000, 73.000]	64.000 [55.000, 73.000]	0.5425
Tumor.Size (median [IQR])	NA	41.000 [26.000, 67.000]	40.000 [26.000, 70.000]	0.7579
Race.ethnicity (%)	Black	5,068 (12.51)	0 (0.00)	<0.0001
	Chinese	493 (1.22)	801 (100.00)	
	Other	3,197 (7.89)	0 (0.00)	
	White	31,769 (78.39)	0 (0.00)	
Sex (%)	Female	14,278 (35.23)	290 (36.20)	0.5934
	Male	26,249 (64.77)	511 (63.80)	
Sequence.number (%)	More	13,360 (32.97)	245 (30.59)	0.1673
	One primary only	27,167 (67.03)	556 (69.41)	
Time [mean (SD)]	NA	39.125 (30.668)	37.527 (30.902)	0.1443
Status (%)	Alive	29,880 (73.73)	583 (72.78)	0.5749
	Dead	10,647 (26.27)	218 (27.22)	
Primary.Site (%)	C64.9-Kidney	38,525 (95.06)	718 (89.64)	<0.0001
	C65.9-Renal pelvis	2,002 (4.94)	83 (10.36)	
Grade (%)	Moderately differentiated	13,895 (34.29)	296 (36.95)	<0.0001
	Poorly differentiated	8,519 (21.02)	239 (29.84)	
	Undifferentiated; anaplastic	3,210 (7.92)	63 (7.87)	
	Unknown	11,708 (28.89)	127 (15.86)	
	Well differentiated	3,195 (7.88)	76 (9.49)	
Laterality (%)	Left	20,044 (49.46)	391 (48.81)	0.0231
	Other	77 (0.19)	5 (0.62)	
	Right	20,406 (50.35)	405 (50.56)	
Pathological (%)	8,312/3: Renal cell carcinoma	7,381 (18.21)	136 (16.98)	0.0079
	8,120/3: Transitional cell carcinoma, NOS	1,088 (2.68)	33 (4.12)	
	8,130/3: Papillary transitional cell carcinoma	995 (2.46)	26 (3.25)	
	8,260/3: Papillary adenocarcinoma, NOS	5,028 (12.41)	76 (9.49)	
	8,310/3: Clear cell adenocarcinoma	21,479 (53.00)	442 (55.18)	
	8,317/3: Renal cell carcinoma, chromophobe type	2,136 (5.27)	49 (6.12)	
	Other(*n* < 1,000)	2,420 (5.97)	39 (4.87)	
T (%)	T1	26,430 (65.22)	481 (60.05)	0.0238
	T2	4,036 (9.96)	100 (12.48)	
	T3	8,075 (19.92)	174 (21.72)	
	T4	1,101 (2.72)	23 (2.87)	
	TX	885 (2.18)	23 (2.87)	
N (%)	N0	36,472 (89.99)	707 (88.26)	0.0142
	N1	2,349 (5.80)	64 (7.99)	
	N2	195 (0.48)	0 (0.00)	
	NX	1,511 (3.73)	30 (3.75)	
M (%)	M0	35,653 (87.97)	678 (84.64)	0.005
	M1	4,874 (12.03)	123 (15.36)	
Surgery (%)	Any nephrectomy in continuity with the resectiont	308 (0.76)	7 (0.87)	0.0034
	Complete/total/simple nephrectomy	3,601 (8.89)	80 (9.99)	
	Local tumor destruction	2,000 (4.93)	51 (6.37)	
	Local tumor excision	852 (2.10)	30 (3.75)	
	No surgery of primary site	7,371 (18.19)	156 (19.48)	
	Partial/subtotal nephrectomy/partial ureterectomy	11,472 (28.31)	198 (24.72)	
	Radical nephrectomy	14,923 (36.82)	279 (34.83)	
Radiation (%)	None/Unknown	38,930 (96.06)	770 (96.13)	0.9922
	Yes	1,597 (3.94)	31 (3.87)	
Chemotherapy (%)	None/Unknown	37,102 (91.55)	708 (88.39)	0.0019
	Yes	3,425 (8.45)	93 (11.61)	
Systemic (%)	None/Unknown	38,183 (94.22)	743 (92.76)	0.0951
	Yes	2,344 (5.78)	58 (7.24)	

### 3.2 Risk factors for distal metastasis

**Figure 2:**
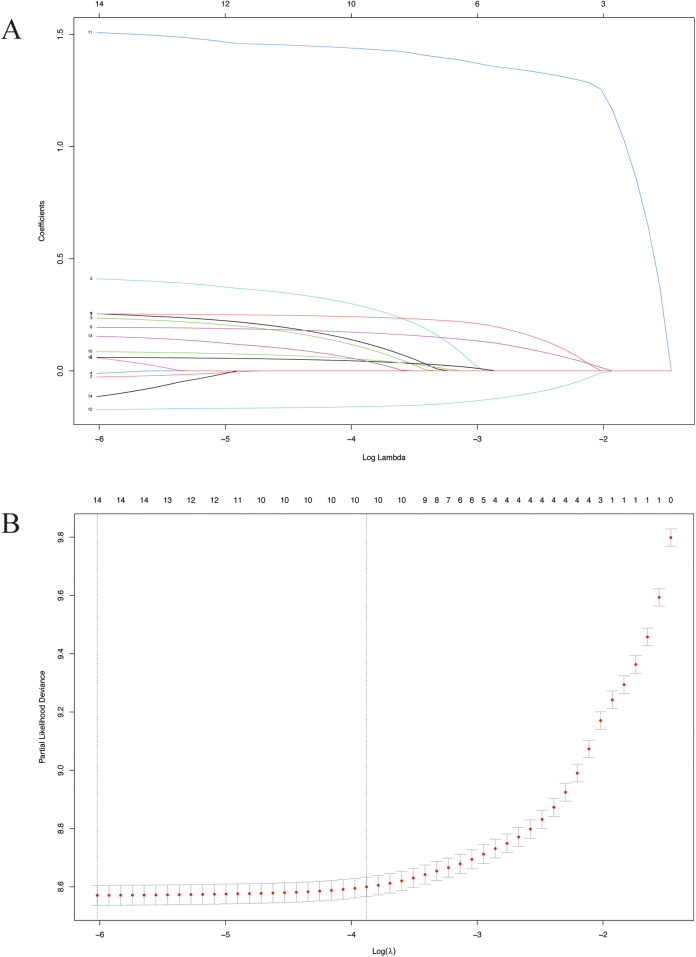
LASSO survival analysis. **(A)** Coefficient profile plots showing how the size of the coefficients of clinical factors shrinks with increasing value of the penalty, with the factors and their regression coefficients selected for the model based on the optimal for the LASSO model. **(B)** Penalty plot for the LASSO model; color error bars indicate standard error. LASSO, least absolute shrinkage and selection operator.

There were 4,874 metastatic patients during the follow-up. To identify independent risk factors for distal metastasis, LASSO (Figures 2A,B), univariate and multivariate regression analyses (Table 2) were utilized in order. The results of univariate analysis demonstrated that age, grade, T-stage, N-stage and tumor size (*p* = 0.007, *p* < 0.001, *p* < 0.001, *p* < 0.001, respectively) were related to distal metastasis. While multivariate analysis further confirmed that these variables can independently influence the distal metastasis of kidney cancer patients.

**Table 2:** Univariate and multivariate logistic regression for distal metastasis of renal carcinoma.

Characteristics	Univariate logistics regression			Multivariable logistics regression		
	OR	CI	*P*	OR	CI	*P*
Age	1.02	1.02–1.02	<0.001	1	1–1.01	0.007
Grade						
Well differentiated	Ref	Ref	Ref	Ref	Ref	Ref
Moderately differentiated	1.77	1.32–2.39	<0.001	1.25	0.92–1.69	0.161
Poorly differentiated	7.18	5.38–9.58	<0.001	2.49	1.85–3.37	<0.001
Undifferentiated; anaplastic	19.95	14.92–26.68	<0.001	3.63	2.67–4.94	<0.001
unknown	19.74	14.88–26.19	<0.001	9.23	6.89–12.36	<0.001
N						
N0	Ref	Ref	Ref	Ref	Ref	Ref
N1	24.08	21.92–26.45	<0.001	8.69	7.81–9.67	<0.001
N2	7.03	5.24–9.43	<0.001	2.18	1.57–3.04	<0.001
NX	6.05	5.4–6.78	<0.001	3.05	2.63–3.53	<0.001
Primary.Site						
C64.9-Kidney	Ref	Ref	Ref	Ref	Ref	Ref
C65.9-Renal pelvis	1.02	0.89–1.17	0.82	NA	NA	NA
T						
T1	Ref	Ref	Ref	Ref	Ref	Ref
T2	6.51	5.89–7.18	<0.001	2.79	2.45–3.18	<0.001
T3	7.61	7.02–8.26	<0.001	3.91	3.5–4.36	<0.001
T4	33.89	29.61–38.79	<0.001	7.8	6.54–9.3	<0.001
TX	39.14	33.73–45.43	<0.001	9.44	7.94–11.22	<0.001
Tumor.Size	1.02	1.02–1.02	<0.001	1.01	1.01–1.01	<0.001

### 3.3 Performance of six machine learning algorithms

**Figure 3:**
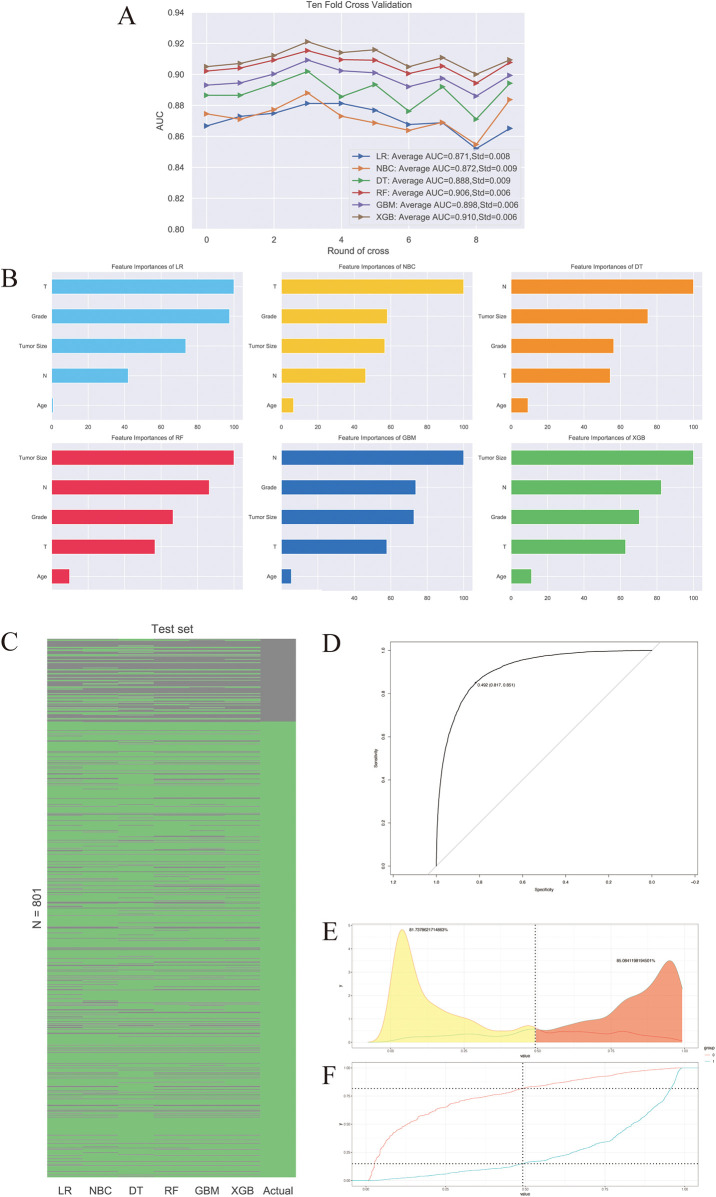
The predictive performance of six machine learning algorithms. **(A)** 10-fold cross-validation of machine learning algorithms. **(B)** Relative importance ranking of features. **(C)** Heat map of accuracy rate of prediction results. **(D)** ROC curve of XGB algorithm. **(E)** Transfer risk density. **(F)** The clinical utility curves (CUCs) of the XGB algorithm.

The predictive performance of six machine learning algorithms was compared via 10-fold cross validation in inner training dataset and ROC analysis in testing dataset. We found XGB had the best performance in predicting distal metastasis in both training (AUC = 0.91) and testing (AUC = 0.851) datasets (Figure 3A). Then T-stage, N-stage, grade, tumor size and age were arranged as per their relative importance in each algorithm (Figure 3B). This order was derived using the built-in gain-based importance metric of the XGBoost algorithm, which measures the average improvement in model accuracy brought by each feature across all trees. The fact that tumor size, N stage, and Grade emerged as the top three most important features suggests that these factors are strongly associated with the occurrence of distant metastasis in our model. This finding aligns well with established clinical knowledge, as larger tumor size, presence of nodal involvement, and higher histological grade are widely recognized as key indicators of aggressive disease and metastatic potential (24). A heatmap showing the predictive accuracy rate of six algorithms and the actual survival status of the testing dataset was displayed in Figure 3C. The cut-off value of XGB algorithm calculated by ROC curve was 0.492 (Figure 3D). The probability density function (PDF) for patients with non-distal metastases was concentrating on a metastasis risk between 0.0 and 0.5, while the PDF for patients with distal metastases was concentrated in a portion representing the metastasis risk (Figure 3E). The clinical utility curves (CUCs) of the XGB algorithm was also conducted, which exhibited the significant clinical utility (Figure 3F).

### 3.4 Establish the nomogram prediction model

**Figure 4:**
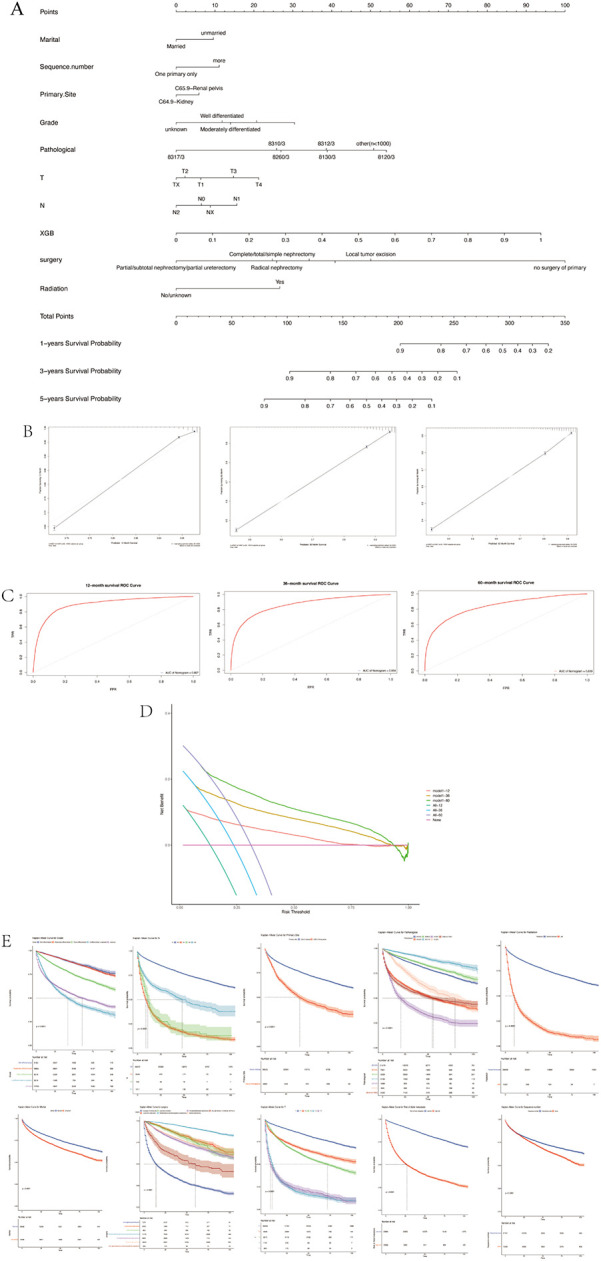
The survival prediction. **(A)** Nomogram. **(B)** The calibration curve. **(C)** ROC curves. **(D)** Decision curve analysis. **(E)** The Kaplan–Meier curves for samples stratified.

Based on the clinicopathological characteristics listed in Table 1, together with the predicted risk of the XGB algorithm, we next employed LASSO Cox analysis to screen independent risk factors to predict survival possibility. Variables including marital status, sequence number, primary site, grade, pathological type, T-stage, N-stage, the calculated risk of XGB, surgical and radiation treatment were incorporated to establish a nomogram to predict the 1-, 3-, and 5-years survival probability (Figure 4A). The ROC and calibration curves of both training and test sets at 1, 3, and 5 years all displayed good consistency between actual and predictive values (Figures 4B,C). And then, the DCA was applied to check the clinical practicability (Figure 4D). The net benefits of the nomogram, in 1-, 3-, 5-year OS prediction, were all superior to the states when all patients survived or none. Furthermore, the Kaplan–Meier curves for samples stratified by above incorporated variables demonstrated (Figure 4E).

## 4 Discussion

The advent of the era of precision medicine has provided more advanced research tools for the development of clinical medicine. AI, as a branch of computer science, is gradually penetrating into the research field of precision medicine through algorithms that simulate human intelligence (25). AI uses intelligent algorithms to mine and extract medical data resources in order to improve the accuracy and effectiveness of clinical treatment. The combination of AI and medical research is one of the key research directions in biomedicine, especially in oncology, which provides a more accurate aid for clinicians' diagnosis and treatment (26). In recent years, AI has been rapidly developed and fruitful results have been achieved in the field of kidney cancer research. As a malignant tumor with high heterogeneity, kidney cancer has various pathological types, and there are obvious individualized differences in patients' treatment effects and clinical prognosis (27). The technologies of AI can provide important support for individualized diagnosis and treatment of kidney cancer.

Over the past two decades, the detection rate of small renal masses has risen significantly, largely attributable to advances in cross-sectional imaging. Partial nephrectomy (PN) is now widely established as the standard treatment for T1 renal parenchymal tumors (28). However, 20%-50% of kidney cancer patients have a distal metastasis or local invasion at initial diagnosis (24). The therapeutic landscape for metastatic renal cell carcinoma has significantly broadened. Interferon alfa, once a conventional option, has been largely superseded by newer agents that demonstrate superior efficacy, including improved response rates and/or prolonged progression-free survival. These advancements comprise antiangiogenic agents directed against VEGF and its receptors, mTOR inhibitors, and immune checkpoint inhibitors, collectively leading to enhanced clinical outcomes and a wider array of therapeutic strategies for this challenging malignancy (5). However, despite these advancements, the treatment of distantly metastatic renal cancer remains a formidable clinical challenge. The early detection of distal metastasis is a crucial measure for clinical decision-making and appropriate management of RCC patients. In this research, a nomogram was built for predicting the risk of distal metastasis in 40,527 kidney cancer patients extracted from the SEER database. We identified ten clinicopathological and demographic features as risk and prognostic predictors, including marital status, sequence number, primary site, grade, pathological type, T-stage, N-stage, the calculated risk of XGB, surgical and radiation treatment.

The impact of marital status on the survival possibility of mRCC was explored previously, which displayed the favorable prognostic effect of marriage on mRCC patients (29–32). Married patients tended to enjoy better survival outcomes than widowed patients in the aspects of both overall survival (OS) and cancer-specific survival (CSS). This may due to the unhealthy lifestyles and scanty financial resources of unmarried patients. Unmarried status was proved to be a barrier for obtaining treatment in mRCC patients (33). While married patients were more likely to receive financial and psychological support from their spouses, so that they can get timely medical care and medication reminders, and avoid psychological distress and depression (31, 34–37).

In our nomogram, we incorporated some vital tumor biological characteristics. The influence of histologic subtype on the metastatic potential of RCC was demonstrated in this study. Indeed, previous studies have found that ccRCC owned the highest metastasis risk, followed by pRCC and chRCC (38). Besides, poorly differentiated RCC generally had inferior prognosis (39–41). With the degree of RCC differentiation from well to poor, the rate of distal metastasis increased (42). This rate can increase by 50% with regional lymph node involvement (43).The tumor size was also an independent risk predictor, with a 2% metastatic proportion for RCC with mean size at 23 mm. When the size of renal neoplasms ≥3 cm, the risk of distal metastasis was higher (44, 45). A linear positive connection can be seen between tumor size and the metastatic rate.

The six applied algorithms are objective, reliable and repeatable in processing big data and can contribute to the inherent paradigm shift in healthcare, thus widely applied in identifying disease progression, improving early diagnosis and predicting survival outcomes. These advantages can facilitate the rational and effective employment of healthcare sources (46). By comparing the AUC values, XGB was found to have the best predictive performance. PDF and CUC further proved its powerful predictions.

Surgical treatment is very crucial for the primary lesion of RCC patients, because the metastasis risk can remarkably increase without nephrectomy (47). Brain metastasis is a typical site of metastasis and its metastatic rate ranged from 2% to 16% in mRCC (48). RCC patients with brain metastasis displayed limited responses to current treatment options with a short median overall survival of only 5–8 months (5, 47, 49, 50). And nonsurgical treatment was a risk factor for brain metastasis from RCC. Bone is another common metastatic site and bone metastasis often occurs in the mid-shaft bone, including osteolytic, osteogenic and mixed lesions. Bone metastasis can lead to skeletal-related events (SRE), such as fractures, hypercalcemia and spinal cord compression, which can have severe influence on patients' quality of life and survival outcomes (51). Although kidney cancer was insensitive to radiotherapy, it can reduce the risk of above SREs (52, 53). According to the findings of Hua et al, radiotherapy can not reduce the all-cause mortality (ACM) and kidney cancer-special mortality (KCSM) of kidney cancer patients with bone metastasis. While for bone metastasis patients, the conclusions about the surgery were discordant. For intermediate-risk patients, the effect of using sunitinib alone was no less than nephrectomy followed by sunitinib (2). While another study proved that ACM and KCSM of patients were markedly improved after surgery. The indications for surgery yet to be explored. And when analyzing the metastatic status and frequencies of renal pelvis cell carcinoma (RPCC), lung and brain were found to be the most and least common metastatic lesions, respectively (54, 55). The influence of the sequence number was also explored. In a previous study, RCC patients with only one primary tumor were more likely to develop bone metastasis. The lack of necessary survival time to form bone metastasis may explain it. While our study demonstrated that more sequence number was related to a worse prognosis.

The ethical implications of applying our predictive model clinically are crucial, particularly concerning patient privacy and data protection in real-world implementation. To address these concerns in potential future applications, we propose the following safeguards: (1) All patient data used by the model will be rigorously anonymized and encrypted both at rest and during transmission. (2) Where feasible, we recommend implementing federated learning techniques that allow the model to be trained and updated across institutions without transferring sensitive patient data. (3) Compliance with Regulations: Any clinical implementation will strictly follow established regulations and other relevant data protection frameworks. (4) Robust access controls and detailed audit trails will be implemented to monitor data usage and prevent unauthorized access.

Although this study included a sufficient number of patients and summarized their information as detailed as possible, the limitations of this study should be notified. First, this was a retrospective study and had inevitable selection bias. Second, apart from the included variables, we may miss some vital biomarkers, genetic mutations, tumor markers, comorbidities, clinical symptoms and treatment responses. Third, we only knew whether these patients received radiation or chemotherapy, but the detailed radiotherapy dose or toxic effects of chemotherapy were unknown, which can also affect the risk prediction. The information about immunotherapy was also lack. Moreover, more external multi-center data are required to verify the accuracy of prediction model.

## 5 Conclusion

The current study identified marital status, sequence number, primary site, grade, pathological type, T-stage, N-stage, the calculated risk of XGB, surgical and radiation treatment as independent prognostic factors of survival possibility in RCC patients. These DM-related risk factors were included to establish a predictive nomogram to screen RCC patients with a high risk of DM.

## Data Availability

The original contributions presented in the study are included in the article/Supplementary Material, further inquiries can be directed to the corresponding authors.
